# Mitochondrial dysfunction and alveolar type II epithelial cell senescence: The destroyer and rescuer of idiopathic pulmonary fibrosis

**DOI:** 10.3389/fcell.2025.1535601

**Published:** 2025-03-31

**Authors:** Suqi Liu, Qian Xi, Xuannian Li, Huaman Liu

**Affiliations:** ^1^ The First College of Clinical Medicine, Shandong University of Traditional Chinese Medicine, Jinan, Shandong, China; ^2^ Six Sections of Geriatrics, Affiliated Hospital of Shandong University of Traditional Chinese Medicine, Jinan, Shandong, China

**Keywords:** idiopathic pulmonary fibrosis, cellular senescence, mitochondrial dysfunction, alveolar type 2 (AT2) cells, senotherapeutics

## Abstract

Idiopathic pulmonary fibrosis (IPF) is a chronic respiratory disease with an unknown origin and complex pathogenic mechanisms. A deeper understanding of these mechanisms is essential for effective treatment. Pulmonary fibrosis is associated with the senescence of alveolar type II epithelial (ATⅡ) cells. Additionally, ATⅡ senescence can lead to a senescence-associated secretory phenotype, which affects cellular communication and disrupts lung tissue repair, contributing to the development of IPF. The role of mitochondrial dysfunction in senescence-related diseases is increasingly recognized. It can induce ATⅡ senescence through apoptosis, impaired autophagy, and disrupted energy metabolism, potentially playing a key role in IPF progression. This article explores the therapeutic potential of targeting cellular senescence and mitochondrial dysfunction, emphasizing their significant roles in IPF pathogenesis.

## 1 Introduction

Idiopathic pulmonary fibrosis (IPF) is an interstitial lung disease of unknown etiology. The disease is chronic, progressive, destructive, and irreversible. The hallmark feature of IPF is the progressive formation and remodeling of lung scarring (Sui et al., 2023). Although the exact course and primary cause of IPF remain unclear, it is now thought that the pathogenesis of the disease may be related to genetic, environmental, viral, or immune factors (Ellson et al., 2014).

Fibrosis is a class of diseases caused by chronic organ damage, characterized by tissue hardening and scarring, and is generally described as excessive pathological deposition of extracellular matrix (ECM) during wound healing (Hinz and Lagares, 2020). Collagen, fibronectin, laminin, and other substances make up the ECM, an intercellular matrix that is essential for healthy tissue healing. Although ECM deposition is an inevitable byproduct of wound healing, if tissue damage persists or recurs, ECM synthesis and remodeling will become uncontrollable, resulting in the formation of persistent fibrotic scars that impair organ function and normal structure (Fischer et al., 2024). Damaged epithelial cells, endothelial cells, and innate fibroblasts are stimulated by injury and undergo transdifferentiation into myofibroblasts, which are capable of strong contraction and matrix formation (Hailiwu et al., 2023). Excessive synthesis and deposition of ECM components lead to tissue structural remodeling and dysfunction, which are caused by myofibroblasts (Bhatt et al., 2024), which are important cellular mediators in the development of fibrosis.

Notably, fibrosis may play a role in the occurrence and spread of cancer. For example, it can improve the mechanical support of tumors, protect tumor cells from immune system attacks, and promote tumor growth by changing local hemodynamics (Tomos et al., 2025). One study showed that the incidence of lung cancer in patients with pulmonary fibrosis was 4.8%–48%, while the incidence in the control group was only 2.0%–6.4%. This difference may be related to the chronic inflammation and lung tissue destruction experienced by patients with pulmonary fibrosis (Kato et al., 2018).

The prognosis of IPF patients is poor, with an average survival of only about 3–5 years after diagnosis (Raghu et al., 2014). Epidemiological studies have shown that people aged 65 years and above have the highest incidence of IPF, and the incidence increases with age (Maher et al., 2021). Compared with people aged 40 years, people aged 70 years and above have a 6.9-fold increased risk of developing the disease (Choi et al., 2018). Therefore, IPF is considered an aging-related disease, and aging is considered an important risk factor for IPF (Wan et al., 2024). Cell growth arrest and reduced replication capacity are hallmarks of aging, and aging makes the lungs susceptible to fibrosis by preventing alveolar progenitor cells from regenerating and cultivating a cellular environment that is conducive to fibrosis (Smith et al., 2022). The primary markers of the link between senescence and IPF include cellular senescence, the senescence-associated secretory phenotype (SASP), and immunosenescence (Salminen, 2025). Alveolar epithelial type II (ATⅡ) cells in IPF patients exhibit pronounced signs of senescence.

Cellular senescence is a key hallmark of aging (López-Otín et al., 2023). In several interstitial fibrosis disorders, senescence—an irreversible cell cycle arrest—is a defining characteristic (Hernandez-Gonzalez et al., 2021). ATⅡ cells are essential for maintaining lung homeostasis (K. Liu et al., 2024). Growing evidence suggests that ATⅡ cell senescence plays a crucial role in the remodeling process of aging-related pulmonary fibrosis. Senescent ATⅡ cells communicate with surrounding cells by secreting SASP factors, which propagate peripheral cellular senescence, promote ECM deposition, disrupt lung structure, impair lung function, and ultimately contribute to the onset of IPF (Yao et al., 2021). In other words, senescent ATⅡ cells activate fibroblasts and myofibroblasts, further exacerbating fibrosis.

A well-established hallmark of cellular senescence is mitochondrial dysfunction (Suryadevara et al., 2024). The term “mitochondrial dysfunction” primarily refers to impaired energy metabolism caused by mitochondrial DNA (mtDNA) damage, disruption of the mitochondrial membrane, inhibition of the respiratory chain, and decreased enzyme activity (Rangarajan et al., 2017). These factors trigger several interconnected damage processes. In addition to being crucial for cellular energy production, apoptosis, and redox balance, mitochondria play a significant role in cellular senescence. Senescence is characterized by mitochondrial alterations, including reduced oxidative phosphorylation (OXPHOS), decreased levels of adenosine 5′-triphosphate (ATP) and nicotinamide adenine dinucleotide (NAD^+^), and an accumulation of reactive oxygen species (ROS), damage-associated molecular patterns (DAMPs), and metabolites from the tricarboxylic acid (TCA) cycle (Martini and Passos, 2023). Many diseases are associated with its dysfunction. Hallmark features of idiopathic pulmonary fibrosis include altered metabolic processes, increased oxidative stress, and reduced cell survival, all of which are caused by mitochondrial dysfunction (Larson-Casey et al., 2020). More and more studies have recognized the importance of mitochondrial dysfunction in aging. It can mediate AT Ⅱ cell senescence, thereby impairing lung tissue regeneration and AT Ⅱ cell differentiation capacity. This ultimately increases the difficulty of maintaining the AT barrier over time and leads to fibroblast barrier activation, which is crucial for the development of fibrotic scars and idiopathic pulmonary fibrosis (Perry et al., 2024).

Transforming growth factor β (TGF-β)/Smad signaling pathway, Wnt/β-catenin signaling pathway, vascular endothelial growth factor (VEGF), fibroblast growth factor (FGF) and platelet-derived growth factor (PDGF) signaling pathways are currently the main focus of research on the pathophysiology and related treatment options of idiopathic pulmonary fibrosis at home and abroad (Claudia et al., 2020). IPF is improved by preventing fibroblast activation and excessive collagen production, reducing cell proliferation and migration, and enhancing blood vessels.

Pirfenidone and nintedanib, which also use this pathway, are both approved by the U.S. Food and Drug Administration (FDA) for the treatment of IPF (Claudia et al., 2020). These drugs do not completely prevent the progressive loss of lung function; instead, they are intended only as palliative therapies for pulmonary fibrosis. They can also cause gastrointestinal problems, photosensitivity, and abnormal laboratory results such as elevated aminotransferases. Because of these negative consequences, IPF patients are less likely to adhere to their medication regimen, which can lead to further disease progression and loss of lung function (Gulati and Luckhardt, 2020). Lung transplantation is the only treatment that can potentially cure IPF, but it is not suitable for most patients (Justice et al., 2019). In addition, the pathophysiology of IPF involves complex immune pathways, irreversible lung tissue fibrosis, and the effects of antifibrotic drugs have their own limitations (Allanore et al., 2024). All of these reasons lead to major challenges in the current treatment of IPF.

**FIGURE 1:**
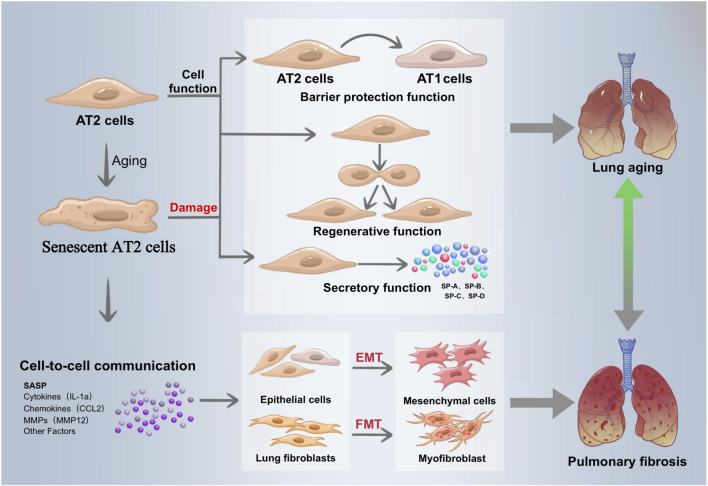
Senescent alveolar type 2 epithelial cells drive the development of IPE. Alveolar epithelial cells are divided into alveolar type 1 epithelial cells (ATI) and alveolar type 2 epithelial cells (ATI2). ATI participates in gas exchange and is found on the alveolar surface. With the ability of develop into ATI in order to restore the damaged ACE barrier, AT2 is a crucial component of the alveolar wall and the primary stem cell in the alveolar repair process. Its functions include barrier protection and regeneration. In addition, AT2 lowers alveolar surface tension control lung immunological activity, secretes a range of alveolar surfactants, and preserves lung function and alveolar structural stability, As people age, their capacity to generate the lung tissue is diminished, their ability to proliferate and differentiate is diminished, their AT2 barrier function is compromised and their ability to replenish alveolar epithelial stem cells is drastically diminished. By secreting SASP, senescent epithelial cells with AT2 cells as their primary constituent encourage the differentiation of epithelial cells into mesenchymel cells (EMT) stimulate neighboring fibroblasts into myofibroblasts (FMT). These action result in extracellular matrix secretion, collagen deposition, and lung tissue remodeling, all of which contribute to the pathological process of pulmonary fibrosis.

Related research is ongoing, and it is crucial to understand how mitochondrial dysfunction and AT II senescence lead to IPF. In order to provide new perspectives for future IPF research and treatment, we systematically summarized the mechanisms of IPF induced by mitochondrial dysfunction in recent years, summarized the pathways of ATII senescence involved in IPF, and elaborated on IPF treatment targeting mitochondrial function and ATII senescence (Figure 1).

## 2 Senescent AT Ⅱ in IPF: Involvement in cellular communication leads to abnormal lung tissue repair

### 2.1 Senescent ATⅡ in IPF leads to abnormal lung tissue repair

**FIGURE 2:**
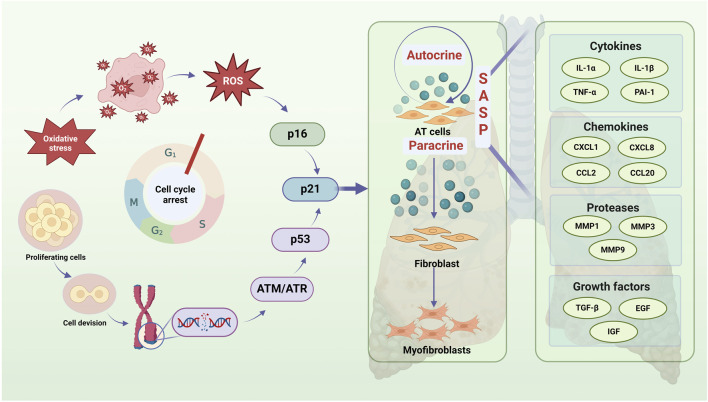
Senescent AT2 contributes to cellular communication, which exacerbates IPF by casing aberrant lung tissue repair. Oxidative stress and DNA damage are major contributions to AT2 cell cycle arrest (particularly G1 cycle) and regenerative failure during IPF. An imbalance between the production of free radical and the cell’s capacity to scavenge them is known as oxidative stress, and its causes ROS buildup and P16 pathway activation. The continuous interference of endogenous and external stressors during cell division and proliferation can cause DNA damage, which sets off DNA damage responses, activates ATM/ATR and other DNA repair pathways, and ultimately influences P53 activation. The aforementioned two routes have the ability to trigger the P21 pathway, impact AT2 cells’ autocrine and paracrine SASP, worsen AT2 cell senescence, and encourage peripheral fibroblasts to become myofibroblasts, all of which have an impact on the onset and progression of IPF. A crucial component of IPF cell communication, SASP contains growth factors, proteases, cytokines, chemokines, and more.

One of the fundamental characteristics of cellular senescence is prolonged or permanent cell cycle arrest. Several factors, including telomere shortening, DNA damage, oxidative stress, senescence gene regulation, and epigenetic alterations, can trigger this process in IPF, ultimately limiting the regeneration of alveolar epithelial progenitor cells (Suryadevara et al., 2024). Single-cell RNA sequencing studies have shown that increased ATⅡ cell senescence in the lung tissues of IPF patients accelerates pulmonary fibrosis by activating pro-fibrotic myofibroblasts through multiple conventional mechanisms (Rubio et al., 2020). The prevailing consensus is that IPF is driven by ATⅡ senescence, which leads to myofibroblast activation and ECM synthesis, resulting in fibrotic scarring and impaired lung tissue repair (Figure 2).

ATⅡ cells play a crucial role in maintaining alveolar structure, stabilizing intrapulmonary function, and serving as the primary stem cells for alveolar repair. They can differentiate into ATⅠ cells to restore the damaged alveolar barrier. According to Witschi’s theory, lung fibrosis originates and progresses due to epithelial damage microfoci. If these microfoci are not repaired in a timely manner, the normal balance between fibroblasts and epithelial cells is disrupted, promoting fibrosis development (Haschek and Witschi, 1979).

When ATⅡ cells undergo senescence, several age-related changes occur in lung tissue, including reduced alveolar epithelial stem cell renewal, impaired ATⅡ function and differentiation capacity, defective lung tissue regeneration, increased expression of senescence markers such as p16 and p21, and altered β-galactosidase activity (SA-β-gal). The failure of senescent ATⅡ cells to maintain the alveolar barrier, coupled with cell cycle arrest, leads to fibroblast activation, proliferation, and collagen deposition, ultimately contributing to fibrotic scarring (Liang et al., 2023).

In this study, we focused on the role of ATⅡ cells in the development of IPF, their contribution to a pro-fibrotic cellular environment, their predisposition to fibrosis, and their regulation of cellular communication through SASP paracrine and autocrine signaling pathways.

### 2.2 Senescent AT II plays a pro-fibrotic role in accelerating IPF development through the involvement of SASP in cellular communication

Atypical lung tissue healing is triggered by the SASP, which primarily consists of growth factors, chemokines, and pro-inflammatory cytokines (Zeng et al., 2024a). Overexpression of SASP has been shown to strongly induce cellular senescence and promote epithelial-mesenchymal transition (EMT) through autocrine secretion (Chilosi et al., 2013). Additionally, SASP regulates the microenvironment in a paracrine manner, stimulating neighboring fibroblasts and myofibroblasts to excessively ECM, leading to lung tissue remodeling and, ultimately, the pathological progression of pulmonary fibrosis. Furthermore, SASP accelerates immune cell senescence and promotes chronic inflammation, which weakens immune function and impairs the clearance of inflammatory factors and senescent cells (Li et al., 2024). This creates a vicious cycle of senescence and inflammation. The immune system dysfunction caused by this cycle is referred to as immunosenescence (Dasgupta et al., 2024).

On one hand, SASP contributes to the progression of IPF through autocrine signaling. Yasunori Enomoto et al. demonstrated that DNA damage induced by bleomycin (BLM) activated p53 signaling in ATⅡ cells, leading to TGF-β-mediated pro-fibrotic gene expression. This initiated a positive feedback loop of TGF-β signaling, which further exacerbated ATⅡ senescence and contributed to IPF development (Enomoto et al., 2023).

On the other hand, SASP secreted by senescent ATⅡ cells can induce senescence in nearby cells through paracrine signaling, playing a crucial role in lung aging and the progression of pulmonary fibrosis. Lehmann hypothesized that the reprogramming of alveolar epithelial cells by SASP components—such as interleukin-6 (IL-6), interleukin-1β (IL-1β), matrix metalloproteinase-12 (MMP-12), chemokine ligand 2 (CCL2), and keratinocyte growth factor—plays a major role in the pathophysiology of IPF. Their study also demonstrated that senescent ATⅡ cells in mice with pulmonary fibrosis secrete higher levels of SASP, which in turn promotes fibroblast-to-myofibroblast transformation, thereby exacerbating fibrosis (Lehmann et al., 2017).

Furthermore, aging ATⅡ cells have been found to promote massive proliferation and activation of fibroblasts and myofibroblasts through the expression of SASP factors such as PDGF, tumor necrosis factor (TNF), endothelin-1, connective tissue growth factor (CTGF), chemokine (C-X-C motif) ligand 12 (CXCL12), and plasminogen activator inhibitor-1 (PAI-1) (Rana et al., 2020). *In vitro* studies by *Rana* T demonstrated that PAI-1 serves as both a marker and mediator of cellular senescence. Notably, PAI-1 knockdown almost completely reversed bleomycin-induced ATⅡ senescence and pulmonary fibrosis in mice (Li et al., 2023).

In conclusion, the autocrine and paracrine involvement of SASP in cellular communication disrupts lung tissue repair, leading senescent ATⅡ cells to drive the development of IPF.

### 2.3 Other pathways

In addition, telomere dysfunction plays a crucial role in the involvement of senescent ATⅡ cells in the development of IPF. A recent study demonstrated that telomerase inactivation due to *TERC* gene deletion not only accelerated ATⅡ cell senescence but also promoted apoptosis and differentiation of ATⅡ cells through both p53-dependent and p53-independent mechanisms (Zhang et al., 2021a). Other studies have shown that telomere dysfunction mediated by *TRF1* deletion can lead to mitochondrial damage and pulmonary remodeling *via* the ECM, with increased expression of senescence markers observed in ATⅡ cells (Naikawadi et al., 2016).

Notably, the accumulation of DNA damage is one of the primary drivers of aging and age-related disorders. Studies have shown that YTHDC1 expression is reduced in both IPF patients and mouse models of pulmonary fibrosis, whereas YTHDC1 overexpression inhibits senescence and mitigates IPF *in vitro* and *in vivo* (Zhang et al., 2024).

These findings suggest that ATⅡ cell senescence disrupts lung tissue repair, thereby accelerating the onset of IPF. They also highlight a promising avenue for further research into the role of ATⅡ senescence in IPF progression. Targeting SASP secretion and intervening in ATⅡ cell senescence could be valuable therapeutic strategies for pulmonary fibrosis, as these approaches may help slow or prevent IPF progression at an early stage.

## 3 The role of mitochondrial dysfunction in alveolar type Ⅱ cell senescence and IPF progression

Mitochondrial dysfunction is a well-established hallmark of cellular aging (Suryadevara et al., 2024). Energy metabolism is one of the key functions of mitochondria. Most of the intracellular ATP is produced by mitochondria, which are also called the “powerhouses of the cell” because they help cells maintain a high ATP/ADP ratio, which is required to thermodynamically drive many metabolic events.

Many conditions, such as oxidative phosphorylation disorders, mtDNA mutations, and abnormal mitochondrial shape and number, can be signs of mitochondrial dysfunction. All of these impairments exacerbate the process of cellular aging and may affect mitochondrial function, including energy production, redox balance, and calcium regulation (Van et al., 2020). In addition, mitochondrial dysfunction disrupts redox homeostasis (Seo et al., 2008), leading to accumulation of cytoplasmic NADH, and a decrease in the NAD/NADH ratio can lead to ATP depletion and cell cycle arrest (Wu et al., 2022).

**FIGURE 3:**
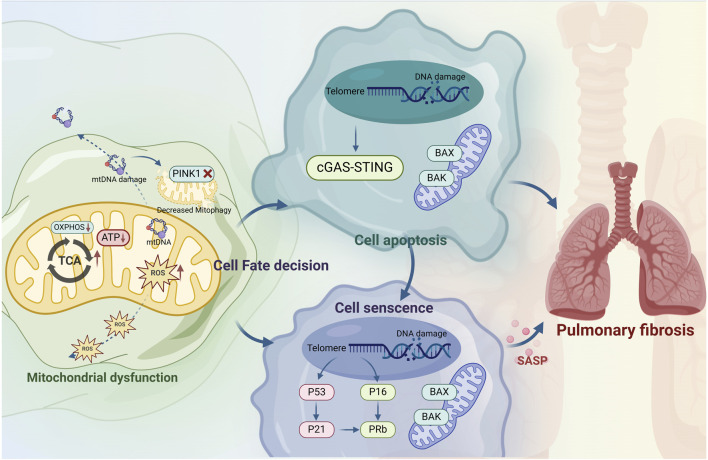
Mitochondrial dysfunction induces idiopathic pulmonary fibrosis through alveolar type II epithelial cell senescence. Mitochondrial dysfunction leads to IPF through apoptosis and senescence. Mitochondrial dysfunction leads to abnormal energy metabolism, decreased mitochondrial ATP production, ROS accumulation, damage to mtDNA, and disruption of PINK1-mediated mitophagy. This process damages chromosomal DNA and activates the p53 pathways, which determines cell fates between apoptosis and senescence, but the specific mechanism is still unclear and may be related to stress. In apoptotic cells, p53 induces mitochondrial outer membrane permeabilization by regulating apoptotic pore formation, which allows cytochrome c release and caspase activation, leading to cell death. Chromosomal DNA damage activates the p53/p21 and p61/pRb pathways, induces cell cycle arrest and exacerbates cells senescence. The upregulation of the pro-survival pathway in senescent cells inhibits the formation of apoptotic pores, resulting in the release of miMOMP, sublethal apoptosis and mtDNA into cytoplasm, and mtDNA fragments are sensed by the cGAS-STING pathway, up-regulating the expression of inflammatory mediators, promoting cell senescence and exacerbating pulmonary fibrosis.

The mitochondrial respiratory chain is essential for maintaining redox balance and intracellular signaling and is the main generator of intracellular ROS (Guan et al., 2024). Mitochondrial dysfunction is the root cause of excessive intracellular oxidative stress and can also affect the functioning of lysosomes, endoplasmic reticulum, and other organelles, promote autophagy, accelerate cell apoptosis, and ultimately lead to cell aging and death (Niforou et al., 2014; Zuk and Bonventre, 2016; Zhou et al., 2022). Additionally, elevated ROS act as endogenous DNA-damaging agents, inducing genetic instability and senescence-related gene alterations (Baranski et al., 2015), which in turn damage alveolar epithelial cells and compromise the epithelial barrier (Figure 3).

In IPF patients, abnormally enlarged and swollen mitochondria have been observed in ATⅡ cells. This is attributed to the disruption of mitochondrial mass maintenance mechanisms, such as mitochondrial biogenesis and mitophagy, which can drive ATⅡ cell senescence. Given their high metabolic demand due to surfactant production, ATⅡ cells are particularly susceptible to mitochondrial dysfunction.

### 3.1 Decreased PINK1-mediated classical autophagy and energy metabolism

ATP production during aerobic respiration is closely linked to the structural and functional integrity of mitochondria. Cells utilize autophagy to remove dysfunctional mitochondria, thereby maintaining mitochondrial homeostasis and normal function (Boyman et al., 2020). However, autophagic activity declines with age (Harrington et al., 2023). Notably, 50% of lung mitochondria are found in ATⅡ cells, making them particularly susceptible to age-related changes such as mitochondrial enlargement, cristae loss, endosome degradation, and reduced respiratory capacity (Sreedhar et al., 2020). Using TEM, Xia observed a significant increase in mitochondrial vacuolization and membrane rupture in senescent ATⅡ cells (Ning et al., 2019), indicating mitochondrial dysfunction and impaired energy metabolism.

Mitochondrial dysfunction has been linked to decreased expression of PTEN-induced putative kinase 1 (PINK1), the primary regulator of mitochondrial homeostasis *in vivo*. Mitochondrial damage disrupts PINK1 translocation, leading to its activation on the outer mitochondrial membrane *via* autophosphorylation. Activated PINK1 recruits and activates the downstream autophagy protein E3 ubiquitin ligase Parkin, which enhances mitochondrial autophagy and mitigates epithelial cell senescence—an essential mechanism for limiting fibrosis (Sosulski et al., 2015). However, maintaining the PINK1-mediated autophagy pathway in senescent ATⅡ cells is challenging (Bueno et al., 2015).

Mitochondrial autophagy reduces SASP factor release and alleviates cellular senescence (Chu et al., 2024). PINK1-mediated mitochondrial autophagy has been identified as a key factor in the pathophysiology of age-related lung diseases such as COPD and IPF (Bueno et al., 2015). Increasing evidence links mitochondrial autophagy and cellular senescence to the progression of IPF in the elderly (Wei et al., 2023a). In mouse lung tissue, PINK1 knockdown in ATⅡ cells resulted in mitochondrial enlargement and dysfunction, impairing mitochondrial autophagy and increasing susceptibility to lung fibrosis (Chu et al., 2024). Thus, the regulation of mitochondrial autophagy plays a critical role in mitigating ATⅡ cell senescence and preventing senescence-associated IPF by preserving mitochondrial homeostasis.

### 3.2 The role of cell apoptosis in alveolar type Ⅱ cell senescence and IPF progression

Groundbreaking research in mitochondrial genetics has demonstrated that mitochondria release cytochrome C, a key component of the electron transport chain (ETC.), to induce apoptosis—a programmed cell death pathway distinct from cellular senescence (Victorelli et al., 2023). Mitochondria play a critical role in apoptosis regulation. Moreover, p53 activation is a pivotal step in aging. In response to various stimuli, p53 upregulates p21 to arrest the cell cycle and subsequently regulates transcriptional programs leading to apoptosis or cellular senescence (Huang et al., 2011). Senescent cells activate the p53/p21 and p16/pRb pathways, characterized by a prolonged DNA damage response (DDR). Additionally, apoptosis is primarily regulated by the Bcl-2 (B-cell lymphoma-2) protein family (Tian et al., 2024). In normal cells, Bcl-2 proteins localize to membrane structures such as the outer mitochondrial membrane (Rasmussen and Gama, 2020), where they interact with Bak and Bax (Bcl-2 Associated X protein) to prevent oligomer formation, maintain mitochondrial membrane integrity, and inhibit cytochrome C release, exerting anti-apoptotic effects (Samuel et al., 2010; Cao et al., 2023).

Mitochondrial dysfunction is a key driver of apoptosis in ATⅡ cells with IPF. The ATM/ATR or AMPK pathway, activated by mitochondrial failure (Anand et al., 2020), phosphorylates p53, stabilizing it and enhancing its transcriptional activity. p53 disrupts the balance of Bcl-2 family proteins by activating pro-apoptotic factors and inhibiting anti-apoptotic proteins, leading to cytochrome C release and the extrusion of mtDNA into the cytoplasm through BAX/BAK pores (Schubert et al., 2024). mtDNA, as a damage-associated molecular pattern, binds to DNA pattern-recognition receptors, triggering the innate immune response *via* the cGAS-STING pathway (Li and Chen, 2018). *In vivo* studies have shown that cGAS-STING activation exacerbates apoptosis in alveolar epithelial cells (Huang et al., 2022). Moreover, persistent mitochondrial damage in IPF may exceed endogenous compensatory mechanisms, leading to chronic accumulation of dysfunctional mitochondria (Kapetanovic et al., 2015).

When ATⅡ cells undergo apoptosis, pro-apoptotic proteins Bak and Bax, initially inhibited by Bcl-2, become activated, undergo conformational changes, and oligomerize on the outer mitochondrial membrane, increasing mitochondrial outer membrane permeability (MOMP) (Subas satish et al., 2024). Excessive MOMP promotes cellular senescence, triggering inflammation and SASP molecule secretion, including IL-6 and IL-8 (Victorelli et al., 2023; Garciaz et al., 2022).

ATⅡ cells from IPF patients exhibit mitochondrial dysfunction and impaired autophagy (Bueno et al., 2015). *In vivo* studies have shown that PINK1-deficient mice develop similar mitochondrial abnormalities in ATⅡ cells, leading to apoptosis and lung fibrosis (Bueno et al., 2015). Additionally, persistent stress can amplify mitochondrial damage (Schuliga et al., 2021). Mitochondrial dysfunction has been reported in IPF, connective tissue disease-associated interstitial lung disease (ILD), and experimental ILD models (Pokharel et al., 2024).

## 4 Targeting cellular senescence and mitochondrial dysfunction in IPF: A promising therapeutic approach

### 4.1 Targeting senescent AT II cells for the treatment of IPF

Senotherapeutics aim to address aging-related health issues by eliminating or suppressing senescent cells. This approach includes two main strategies: senolytics and senomorphics (Table 1). Senolytics induce apoptosis in senescent cells by targeting anti-apoptotic pathways. For example, ABT-263 (Zhu et al., 2016) and ABT-737 (Yosef et al., 2016) bind to BCL-2, BCL-XL, and BCL-W, triggering apoptosis in senescent cells. Another senolytic, ABT-263, specifically inhibits BCL-2 and BCL-XL, effectively eliminating senescent cells. *In vitro* and *in vivo* studies confirm its anti-aging and anti-fibrotic properties, suggesting potential for treating age-related fibrotic diseases. Dasatinib (D) and quercetin (Q), when combined (D + Q), selectively induce apoptosis in senescent human cells without affecting non-senescent cells. This combination was the first identified anti-aging drug and has shown promise in improving age-related conditions in mice and in limited human trials (Justice et al., 2019).

**TABLE 1:** Strategies targeting senescent AT II cells for the treatment of IPF.

Targets of Action	Drug name	Verified path	PubMed ID
Senescent cells	ABT-263	vitro	26,711,051
		vivo and vitro	34,318,888
	ABT-737	vivo and vitro	27,048,913
	Dasatinib	clinical trials	30,616,998
	Quercetin		
	Roxithromycin	vivo and vitro	33,654,217
	SSK1	vivo and vitro	32,341,413
	Procyanidin C1	vivo and vitro	34,873,338
SASP	Rapamycin	vivo and vitro	28,371,119
	Resveratrol	vitro	28,329,136
	Metformin	vivo and vitro	26,990,999
		clinical trials	
	Aspirin	vitro	16,039,999
	Rutin	vivo and vitro	37,475,161

Recent studies suggest that roxithromycin inhibits senescence through a NOX4-dependent mechanism, making it a potential treatment for IPF (Zhang et al., 2021b). However, researchers caution against its immediate use as a senolytic due to concerns about antimicrobial resistance. Instead, its properties could inform the development of future senolytic drugs. Another promising compound, senescence-specific killing compound 1 (SSK1), is activated by β-gal and removes senescent cells *via* p38/MAPK signaling. *In vivo* and *ex vivo* studies demonstrate its ability to alleviate IPF, reduce inflammation, and decrease senescence-related gene expression (Cai et al., 2020). Additionally, research on grape seed extract led to the successful isolation of proanthocyanidin C1, which has been shown in cellular and animal studies to effectively eliminate senescent cells and potentially extend lifespan.

Senomorphics, a newer anti-aging approach, mitigate the harmful effects of senescence by inhibiting SASPs rather than directly eliminating senescent cells. Representative senomorphics include metformin, rapamycin, and resveratrol (Wang et al., 2017; Menicacci et al., 2017; Noren hooten et al., 2016). Other potential senomorphics include aspirin, NF-κB inhibitors, p38 MAPK inhibitors, JAK/STAT inhibitors, ATM inhibitors, and statins (Bode-Böger et al., 2005). Unlike senolytics, senomorphics primarily target SASP to prevent the paracrine/autocrine spread of senescence to neighboring and distant cells.

Rapamycin, for example, has demonstrated therapeutic effects on IPF in both *in vivo* and *ex vivo* studies by inhibiting the mTOR pathway, thereby reducing SASP production and inflammation while slowing cellular senescence (Chrienova et al., 2022). Similarly, rutin, a natural compound, has been identified as a potential senomorphic that suppresses SASP expression and may be used to treat aging-related diseases (H. Liu et al., 2024).

By targeting the initial senescent cells, senomorphics not only prevent senescence from spreading but also disrupt the cycle that promotes further accumulation of senescent cells. While generally less potent than senolytics, natural polyphenols are gaining popularity due to their low toxicity and availability.

### 4.2 Targeting dysfunctional mitochondria for the treatment of IPF

The complex nature of mitochondrial dysfunction and its elusive phenotypic thresholds make it challenging to fully understand its role in disease. However, mitochondria remain key targets for therapeutic intervention. Mitochondrial failure contributes to pulmonary fibrosis by driving ATⅡ cell senescence. Given this, targeting mitochondrial dysfunction offers a promising strategy to treat or delay IPF progression. Advances in mitochondrial biology have led to new therapeutic approaches, though human clinical trials remain limited, highlighting the need for further research. Potential treatments include antioxidants and drugs that enhance mitochondrial ATP production, reduce oxidative stress, and improve mitochondrial function (Wal et al., 2024). These strategies range from dietary interventions addressing nutritional deficiencies to pharmacological therapies that modulate mitochondrial dynamics, boost biogenesis, and mitigate oxidative damage (Table 2).

**TABLE 2:** Strategies targeting mitochondrial dysfunction for the treatment of IPF.

Targets of Action	Drug name	Verified path	PubMed ID
oxidative stress	Mdivi 1	vivo	23,239,023
	P110	vivo	23,239,023
	Senegenin	vivo	38,929,114
	TH5487	vivo	38,929,114
	MitoQ	vivo	16,829,229
Mitophagy	TET	vivo and vitro	38,438,063
	Tetrandrine	vitro	38,438,063
	Naringin	vivo	36,688,958
	Spermidine	vivo and vitro	30,154,567
	Resveratrol	vivo and vitro	38,865,904
	Urolithin A		
	Magnokiol		
cGAS/STING	Urolithin A	vivo and vitro	30,154,567
	Przewalskin	vivo	38,237,513
	Harmine	vivo	38,924,867
	BAI 1	vivo	38,168,624
	MitoTam	clinical trials	29,786,070

Reducing mitochondrial oxidative stress is a critical therapeutic goal. Inhibitors of mitochondrial fission play a key role in achieving this (Qi et al., 2013; Yang et al., 2024; Ko et al., 2021). Mdivi-1, for example, counteracts excess ROS by blocking Dynamin-Related Protein 1 (DRP1) GTPase activity, improving endothelial function and reducing inflammation in animal models (Qi et al., 2013). Similarly, the peptide P110 enhances mitochondrial function by inhibiting DRP1 (Qi et al., 2013). Senegenin has been shown *in vivo* to prevent oxidative stress-induced epithelial cell senescence and reduce lung fibrosis by modulating the Sirt1/Pgc-1α pathway (Zeng et al., 2024b). In BLM-induced lung fibrosis models, TH5487 reduced oxidative stress, promoted PINK1/Parkin-mediated mitophagy, and alleviated mitochondrial dysfunction. Additionally, clinical research indicates that MitoQ scavenges free radicals, protecting cells from oxidative stress and enhancing cellular function.

Mitochondrial autophagy (mitophagy) plays a vital role in mitigating pulmonary fibrosis caused by ATⅡ cell senescence. Tetrandrine (TET) has been shown to reduce lung inflammation and fibrosis by regulating mitophagy through the PINK1-Parkin signaling pathway (Chu et al., 2024). In MLE-12 cells, TET rescued impaired BLM-induced mitophagy by preventing the reduction of autophagy-related protein expression, while PINK1 gene knockdown abolished its effects (Chu et al., 2024). Naringenin has also been found to regulate mitophagy and alleviate pulmonary fibrosis *via* the ATF3/PINK1 pathway (Wei et al., 2023b). Several natural compounds, including spermidine, resveratrol, and urushiol A, support mitochondrial integrity by stimulating mitophagy and promoting biogenesis (Palikaras et al., 2018).

The cGAS/STING pathway is closely linked to aging and lung fibrosis, though its precise role remains unclear. Recent studies indicate that urushiol A-induced mitophagy reduces free cytoplasmic mtDNA activation of cGAS/STING, improving mitochondrial function. Additionally, homopalol enhances SIRT3 deacetylation activity, activating SOD2’s antioxidant function and OGG1-mediated DNA repair. This modulation of the cGAS/STING pathway helps prevent fibrosis and cellular senescence (Wu et al., 2024). Other compounds, such as purple salvia terpene ether and salvia divinorum extract, have been shown to reduce BLM-induced lung fibrosis by inhibiting TGF-β1 signaling, oxidative stress, and collagen deposition (M. Zeng et al., 2024). Harmine has been confirmed *in vitro* to prevent pulmonary fibrosis by regulating DDR-associated genes and activating the TP53-Gadd45α pathway (Gong et al., 2024).

Targeting inflammation linked to mitochondrial outer membrane permeabilization (miMOMP) may also be beneficial. For example, BAX inhibitor BAI-1 reduces mitochondrial BAX and BAK nanopores, lowering systemic inflammation and extending healthy lifespan in aged mice (Victorelli et al., 2023). Additionally, MitoTam, a mitochondria-targeted tamoxifen currently in clinical trials, has been shown to induce apoptosis in senescent cells and reduce senescence markers (p21 and p16) in the kidneys and lungs of aged mice (Hubackova et al., 2019).

Gene replacement therapy and gene editing technologies offer potential solutions for inherited mitochondrial disorders, though a deeper understanding of the mitochondrial genome is required. Combination therapies have also gained attention; for instance, combining MitoQ with senolytics may enhance mitochondrial function while reducing senescent cell burden.

## 5 Conclusions and prospects

Although aging and IPF—a disease primarily affecting the elderly—are closely linked, cellular senescence in IPF differs significantly from normal physiological aging (Hagood, 2014). Normal senescence is a gradual, systemic decline, whereas in IPF, cellular senescence is rapid, localized, and pathologically driven, leading to alveolar epithelial failure and fibrosis (Confalonieri et al., 2022). In IPF, ATⅡ cells are the primary targets of senescence, with mutation-driven, SASP-mediated, and microenvironmental consequences that disrupt the alveolar barrier and activate fibroblasts, ultimately causing irreversible fibrosis (Confalonieri et al., 2022; Lei et al., 2024). In contrast, normal aging results in a slow decline in organ function and increased susceptibility to age-related diseases but is not inherently fatal. Therapeutic strategies for aging include mTOR inhibitors, antioxidants, anti-aging therapies, and calorie restriction, whereas IPF-specific treatments focus on antifibrotic agents and telomerase activation (Cătană et al., 2018). While approved antifibrotic drugs like pirfenidone and nintedanib slow IPF progression, they have side effects and cannot reverse lung function decline. Therefore, research on IPF aging could enhance pulmonary fibrosis management while offering new insights into anti-aging therapies.

Senolytic drugs targeting tumorigenic pathways have emerged as a promising class of therapies, though their long-term optimization remains challenging. Additionally, the chronic lung damage and inflammation in IPF may share mechanistic links with cancer, offering potential directions for anti-aging drug development. Targeting the “two-way vicious cycle” between mitochondrial dysfunction and ATⅡ senescence may provide precision therapies for IPF. This cycle involves mitochondrial damage triggering senescence *via* ROS and energy depletion, while senescent cells further impair mitochondria and activate fibroblasts through SASP. While most evidence suggests ATⅡ senescence promotes fibrosis, some SASP factors may inhibit collagen production, highlighting a complex, microenvironment-dependent effect.

Future research should explore novel drugs targeting this pathway to treat pulmonary fibrosis. Investigating the clinical potential of compounds that have shown efficacy in animal and cellular models could accelerate the development of effective IPF therapies. Mitochondria-targeted approaches align with precision medicine, offering tailored treatments that mitigate ATII cell senescence while minimizing adverse effects. However, the roles of many naturally occurring compounds in modulating mitochondrial autophagy, ATP production, and oxidative stress remain unclear. By targeting multiple pathways associated with mitochondrial dysfunction, these treatments could help counteract fibrosis progression.

Implementing a personalized medical approach requires clinically relevant biomarkers. A robust biomarker panel, monitored longitudinally, could facilitate early detection of treatment responses in clinical trials. However, challenges remain, including variability in staining techniques, sensitivity issues due to small sample sizes, and the need for independent validation. Most research is still limited to preclinical models, necessitating further human trials to assess the efficacy of senescence-targeting and mitochondrial therapies in IPF. Additionally, substances that show promise in treating fibrosis-like conditions in other organs may not necessarily be effective against pulmonary fibrosis. Variability in experimental methods, reagents, and models further complicates research outcomes. The interactions between cellular senescence, mitochondrial dysfunction, and other disease factors remain poorly understood, and a major hurdle in IPF treatment is the lack of therapies that simultaneously target both ATⅡ cell senescence and mitochondrial dysfunction.

Advancements in technology are deepening our understanding of IPF pathogenesis, which could optimize therapeutic strategies. As research progresses, new treatments targeting key molecular pathways are being developed, and clinical trials are gradually advancing. Gene therapies related to aging and pulmonary fibrosis are also emerging, propelled by advances in genetics and gene editing. Ultimately, IPF treatment is evolving toward greater precision and personalization, aiming to minimize side effects and improve patients’ quality of life. With continued drug development and ongoing clinical trials, there is reason to be optimistic about the future of IPF treatment.
